# Impact of *Working Together* for adults with autism spectrum disorder: a multifamily group intervention

**DOI:** 10.1186/s11689-021-09395-w

**Published:** 2021-10-08

**Authors:** Leann Smith DaWalt, Emily Hickey, Rebekah Hudock, Amy Esler, Marsha Mailick

**Affiliations:** 1 Waisman Center, University of Wisconsin-Madison, 1500 Highland Ave, Madison, WI USA; 2 Department of Pediatrics, University of Minnesota, Minneapolis, USA

**Keywords:** Autism spectrum disorder, Adulthood, Multi-family group psychoeducation, Behavior problems

## Abstract

**Background:**

Adults with autism spectrum disorder (ASD) have lower engagement in their communities, higher rates of unemployment/underemployment, and continued difficulties with challenging behavior compared to their neurotypical peers. Multi-family psychoeducation emphasizes education and problem-solving with the goal of improving these outcomes for the individual with the disability.

**Methods:**

Using a randomized waitlist control design, the present study evaluated a multi-family group psychoeducation intervention, *Working Together,* for adults on the autism spectrum without intellectual disability (*n* = 40). Five waves of data were collected at 3-month intervals. In this design, families in the intervention condition participated in intervention during the 6 months between baseline and time 3 data collection; the waitlist control condition received the intervention immediately after the time 3 data collection. We compared these two conditions, intervention group (*n* = 20) vs waitlist control group (*n* = 20), on key outcomes for the adults with ASD: engagement in work-related activities, engagement in meaningful activities, and behavior problems.

**Results:**

Results indicated medium to large effect sizes associated with the *Working Together* intervention across key outcomes, including adults on the spectrum experiencing significant increases in meaningful activities and decreases in internalizing problems. Although increases in work-related activities were not statistically significant, an observed one-half of a standard deviation difference from before to after the intervention indicated clinically significant change. We also found maintenance of the treatment effect through 6 months post-treatment for the intervention group and replication of the treatment effect within the control group after they received the intervention.

**Conclusion:**

*Working Together* is a promising multi-family group psychoeducation intervention designed to improve functioning during adulthood. These findings highlight the need for more intervention services research during adulthood and specifically the need for family-centered supports.

**Supplementary Information:**

The online version contains supplementary material available at 10.1186/s11689-021-09395-w.

Adults with autism spectrum disorder (ASD), including those without co-occurring intellectual disability (ID), have strikingly poor adult outcomes compared to their neurotypical peers including lower engagement in meaningful activities, high rates of unemployment/underemployment, and continued difficulties with challenging behavior [16, 28, 37, 41, 53, 57, 70, 76–78, 102]. Entitlement to many formal services ends for these individuals at their entry into adulthood, resulting in long-term reductions in behavioral, social, and transportation supports that may be necessary to continue to engage in the community and live a full life [50]. Of particular concern, there is evidence that the gains made in behavioral functioning during adolescence may plateau or even decline after these individuals exit high school [86, 95], suggesting adulthood as a period of heightened risk. Given the rapid increase of autism diagnoses since the 1990s [33, 55], more adults than ever before have an ASD diagnosis, creating a pressing need for interventions that promote positive outcomes during this life stage. The present study focused on evaluating a new multi-family group psychoeducation intervention, *Working Together,* designed to increase engagement in meaningful activities, both paid and unpaid, and improve behavioral functioning (i.e., reduce challenging behavior) for *disengaged* adults with ASD without co-occurring ID. In this study, we defined disengaged as those who have less than 10 h/week of paid employment/educational programming, consistent with prior research in similar samples [91, 97]**.**

## Engagement in work, meaningful activity, and behavioral functioning

National survey data reveal that 42% of adults with ASD are never employed at any point during their 20s [71]. Similarly, in a community-based sample of adults with ASD, Taylor and Seltzer [95] found that only 18% of the sample had competitive or supported employment. Further, adults with ASD *without* ID may be at particular risk for low engagement in work activities. In a sample of young adults with ASD, 56% of the adults spent time in sheltered workshops or day activity centers; yet, young adults with ASD *without* ID were three times more likely to have no daytime activities compared to adults with ASD who had ID. Specifically, over 25% of the adults in the sample *without* ID had no daytime activities of any kind compared to only 8% of young adults with ID, suggesting a disparity between those with and without ID in the availability and/or appropriateness of adult services [95]. A follow-up study found that even when individuals with ASD without ID had employment at one time point, few maintained these activities over time, and this was particularly true for women [91].

Adults with ASD are also at-risk for challenges with co-occurring emotional and behavioral functioning, particularly in the area of internalizing behavior problems. For example, lifetime estimates for individuals with ASD range as high as 23–37% for depression and 27–52% for anxiety disorders [21, 38, 88], compared to rates of 10% and 9% in the general population [21]. There is also evidence to suggest that engagement in work may be linked with emotional and behavioral functioning. For example, using two time points of data separated by 5.5 years, Taylor et al. [92] examined the directionality of associations between vocational independence and emotional and behavioral functioning for 153 adults with ASD and found that adults who initially had more vocational independence had greater subsequent improvement in autism symptoms, maladaptive behavior, and daily living skills. The opposite direction of effects (earlier behavioral measures to subsequent vocational independence) was not statistically significant. Thus, behavioral difficulties might be alleviated to some extent through vocational engagement.

Although less studied, the same patterns may be true for other, non-paid activities that are meaningful and important to the adult with ASD. Limited additional research has shown that increased participation in leisure activities predicts lower behavioral problems [31] and independent engagement in daily activities such as self-care, meal prep, and household tasks [39]. Importantly, this engagement in activities of daily living has also been linked to higher quality of life in adults with ASD [39]. It is likely that under-stimulation (i.e., lack of engagement) may magnify challenging behaviors (e.g., apathy, boredom, depression, loneliness and agitation) as has been found in research in populations of individuals with dementia [61] and that engagement in personally meaningful activities may improve psychosocial outcomes for adults with ASD. Taken together, these studies point to a pressing need for interventions to support both the engagement of adults with ASD in meaningful vocational and non-vocational activities as well as their behavioral health.

## Role of the family

Parents of individuals on the autism spectrum are often significant sources of support across adult life and bidirectional influences between adults with ASD and their family members are well-documented [6, 13, 32]. Our longitudinal work among families of adolescents and adults with ASD has demonstrated that high levels of emotional intensity in the home can lead to increases in behavior problems and autism symptoms over time [6, 32]. In contrast, when parents of youth and adults with ASD are warm and positive toward their children, the behaviors and autism symptoms of their children abate ([84] [101, 102];). Given the centrality of the family in the lives of adults with ASD, it is strategically advantageous to involve the family in change processes. However, most services available for individuals with ASD during adulthood do not involve family members as partners and there are no empirically based interventions designed for families during their son or daughter’s adulthood [66, 74, 90].

Multi-family group psychoeducation is a well-developed intervention approach with proven efficacy among families of individuals with psychiatric conditions such as schizophrenia [24, 59] and mood disorders [19, 60]. Although the content and components of psychoeducation interventions vary by study and type of condition or disability, they have key elements in common: *The intervention involves weekly group sessions wherein multiple families are provided with relevant education and resources related to the disability or mental health condition as well as training in, and activities for, practicing problem-solving. This process involves both the person with the condition and at least one family member.* Multi-family group psychoeducation emphasizes education and problem-solving with the goal of improving outcomes for the individual with the disability. In our prior intervention work with adolescents with ASD, participation in multi-family group psychoeducation was associated with significant increases in social engagement (e.g., hanging out with friends, calling/texting) for the adolescents with ASD [22] as well as improvements in the parent-child relationship [86]. The present study examined how multi-family group psychoeducation may improve engagement in work-related activities and other meaningful activities and reduce behavioral challenges for adults with ASD.

## Present study

The present study examined the effects of *Working Together*, a multi-family group psychoeducation intervention for adults with ASD without ID, designed by the first author, using a randomized waitlist control design. Five waves of data were collected at three-month intervals (time 1 = baseline, time 2 = 3-month follow-up, time 3 = 6-month follow-up, time 4 = 9-month follow-up, and time 5 = 12-month follow-up). In this design, families in the intervention condition participated in *Working Together* during the 6 months between baseline and time 3 of data collection; the waitlist control condition received the intervention immediately after the time 3 data collection. We compared two conditions, intervention group vs. waitlist control group, on key outcomes for the adults with ASD: engagement in work, engagement in meaningful activities, and behavior problems.

Our first and primary research question was the following: is there a difference in change from baseline to post intervention (measured at the time 1, time 2, and time 3 points of data collection) for adults in the intervention compared to adults in the waitlist control condition on the three key outcome measures? The three primary outcomes in the current study were (1) engagement in work, (2) engagement in meaningful non-work activities, and (3) behavior problems. We hypothesized that individuals who received the intervention would have greater improvements in these three outcomes than individuals in the waitlist control condition. We also explored maintenance and replication of the treatment effects. Specifically, for our second question, we asked if there was maintenance of the intervention effect for the intervention group during the 6-month period when they were no longer receiving any intervention (time 3, time 4, and time 5 points of data collection). We hypothesized that there would be maintenance of intervention effects as evidenced by lack of decline. Next, for our third question, we asked if there was significant change in adult outcomes for the waitlist control group after receiving the intervention (time 3, time 4, and time 5 waves of data collection); significant improvements in the waitlist control group would reflect a replication of the treatment effect in a separate cohort. We hypothesized that the waitlist control group, when given the intervention, would show similar patterns of change in the three outcomes of interest as the intervention group.

## Methods

### Participants

**Fig. 1:**
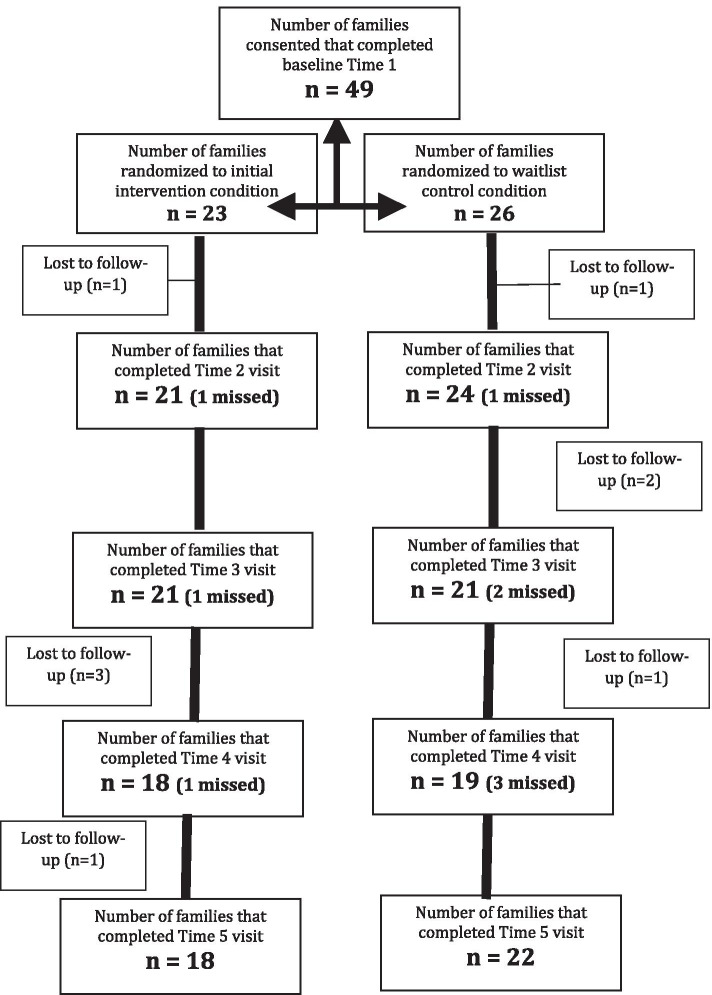
Consort diagram note: Number of families who missed a particular timepoint but remained in the study is noted in parentheses

Families of adults with ASD (*n* = 49) were recruited from two midwestern states through local autism groups, clinics, and university research registries. In order to better understand the ASD symptomology and phenotype of our sample, adults with ASD were assessed using the Childhood Autism Rating Scale, Second Edition (CARS-2 [81];), which has been validated extensively in adult samples and was designed to build on its predecessor (CARS [80];) by adding an additional rating scale intended to identify individuals with average IQ [81, 98], to characterize current autism symptoms. The *Wechsler Abbreviated Scale of Intelligence* (WASI-II [100];) was used to describe the intellectual abilities of the adults in the current sample, and the Waisman Activities of Daily Living [54] was used to characterize daily living skills. Inclusion criteria for the present study were (1) the adult with ASD was 18 to 30 years of age, (2) the adult had a full-scale intelligence quotient equal to or greater than 70, (3) confirmation of the adult’s independent medical diagnosis or educational label of autism spectrum disorder using the Social Communication Questionnaire-Lifetime (SCQ [72];), (4) adult co-resided with parent(s), and (5) adult spent less than 10 h/week in employment/educational activities. As shown in Fig. 1, following baseline data collection (time 1), families were randomly assigned to the intervention condition (*n* = 23) or waitlist control condition (*n* = 26). For our first and primary research question, we included only individuals with complete data across the first three waves of data collection (times 1, 2, and 3), resulting in an analytic sample of 40 families (20 intervention, 20 control).

In the current sample, adult-family member dyads were recruited; many families involved more than one parent or parent figure in the study. In these cases, based on family preference, one parent/parent figure was designated as the primary respondent for research purposes. It should also be noted, however, that we defined “family” broadly and could include a parent, aunt/uncle, grandparent, sibling, etc. Of the primary parent/caregiver respondents in our analytic sample, 87% were female and the majority (92%) was non-Hispanic White; all but two primary respondents were parents of the adult with ASD. The median level of educational attainment of the primary parent/caregiver was a completed bachelor’s degree, with 65% reporting a bachelor’s degree or higher. The families’ household incomes ranged from under $20,000 to over $160,000 dollars per year (pre-tax income in 2016), with a median of $80,000. The adults in our analytic sample ranged in age from 18 to 30 years (*M* = 21.77; SD = 2.94) and their average age of diagnosis was 10.38 years (SD = 5.71; range 2–22 years); 65% were male and almost 90% were White.

As presented in Table 1, there were no statically significant differences between the intervention and control groups at baseline in terms of parental sex, educational attainment, or family income, or in the adult with ASD’s variables of sex, age, race/ethnicity, receiving Medicaid, autism symptoms, daily living skills, or intelligence. There also were no differences at baseline in number of types of behavior problems, which was an outcome variable of interest.

**Table 1:** Background variables at time 1 for intervention (*n* = 20) and control (*n* = 20) groups

	Intervention	Control	*F* or chi-squared
Parent variables			
% Female	85	95	1.12
Educational attainment			5.61
%High school	5	0	
%Some college	40	24	
%Bachelor’s degree	35	29	
%Graduate degree	20	47	
Average family income	$75,000	$78,000	0.08
Adult variables			
% Male	65	65	0.42
Age	*M* = 21.63 (SD = 2.73)	*M* = 21.94 (SD = 3.26)	0.09
% Non-Hispanic White	79	94	0.20
% Receiving Medicaid	45	40	.10
Number of type of behavior problems	*M* = 3.65 (SD = 1.81)	*M* = 3.55 (SD = 1.73)	0.03
Autism symptoms	30.05 (4.47)	29.26 (5.30)	.24
Daily living skills	*M* = 25.47 (SD = 4.85)	*M* = 24.73 (SD = 3.54)	0.30
IQ	*M* = 106.79 (SD = 12.90)	*M* = 110.65 (SD = 16.82)	.64

### Procedures

The study was approved by the University of Wisconsin-Madison and University of Minnesota Institutional Review Boards and all participants provided written informed consent before beginning the study. Data were collected from parents and adults at five time points separated by approximately three months: baseline, 3, 6, 9, and 12 months (times 1–5, respectively). For all waves of data collection, parents participated in a standardized interview and completed self-administered questionnaires regarding the behaviors and activities of their son or daughter; we have successfully employed these instruments and process in our prior work with families of adolescents and adults with ASD (e.g., [22, 101, 102]). Adults also participated in a separate structured interview at each time point to answer questions related to well-being and daily activities. The interviews and questionnaires were identical at each wave of data collection with three notable exceptions: (1) the baseline parent questionnaire included demographic questions, (2) the baseline adult assessment included cognitive and autism symptom testing, and (3) data collection following completion of the intervention included questions related to satisfaction with the intervention.

Following the completion of the baseline data collection (time 1), families in the intervention condition participated in the *Working Together* intervention program (described below); families in the waitlist control condition participated in the intervention following the time 3 data collection.

The *Working Together* program was developed through an iterative process that involved integrating existing research with stakeholder input. Specifically, we created a detailed curriculum based on our longitudinal research and past intervention work, conducted a series of focus groups with key stakeholders (e.g., individuals with ASD, family members, service providers), and implemented a feasibility pilot in order to gain families’ perspectives on the content and process of the intervention. Throughout the development of the intervention, we partnered with an advisory board (comprised of 1 adult with autism, 4 family members, and 2 service providers) to ensure that the intervention would be community-informed. The research team also included two adults with ASD and one parent of an individual with ASD who were involved in material creation, intervention implementation, and evaluation. The resulting *Working Together* intervention involved 2 individual family joining sessions, 8 weekly parent and adult group sessions, 3 monthly booster sessions, and ongoing resources and referrals.

Joining sessions, attended by the adult with ASD and their parent/caregiver, lasted approximately 1 h. The purpose of the joining sessions was to build rapport and partnership and to clarify unique family goals for the program through the use of activities such as a family timeline and ecomap for each family member involved. An ecomap is a graphic map portraying sources of stress and support across an individual’s ecological system and the connections the individual has to each [34] that has been successfully used as an assessment tool in our prior work with youth with ASD [49]. Goals identified by adult with ASD fell into 5 major areas: (1) employment goals such as “communicate with a job coach,” (2) social goals such as “call or text a friend,” (3) independence goals such as “research options for living on my own” or “get a driver’s license,” (4) health goals such as “make a good food choice,” and (5) personal goals such as “get a pet.” Each family was allowed to include additional family members of their choosing to attend the joining sessions (e.g., some families invited a sibling or grandparent to attend), although most joining sessions were comprised of participating parents and adults with ASD.

After families completed joining sessions, the weekly group sessions were held at the same time and place for both adults with ASD and parents/caregivers, but in different rooms such that the adults with autism were only in group sessions with other autistic adults and parents were only in group sessions with other parents. Group sessions lasted approximately 1.5 h and were comprised of 4–7 families per group. Intervention group sessions for adults and their parents involved education on a variety of topics relevant to ASD as well as guided practice in problem-solving (session topics and goals are presented in Table 2). For example, participants were coached on how to use SMART goal-setting strategies [63], how to create and maintain a “goal planner,” how to effectively use a 4-step method of problem solving (1, define problem; 2, list all possible solutions; 3, consider pros and cons; 4, choose the best solution), and how to identify fulfilling activities to practice problem-solving as a family.

**Table 2:** Overview of session topics and goals

Session	Topic	Adult group goals	Parent group goals
Group meeting 1	Introduction	Meet other adults on the spectrum Find shared interests [68] ^a^	Meet other families Learn about developmental course of ASD ([75] [76] [77];);^a^
Group meeting 2	Goal setting and problem solving	Learn about goal setting Write personal goals that are realistic and measurable	Learn about educational/training services and goal setting [95] ^a^ Learn and practice problem-solving method
Group meeting 3	Coping strategies and problem solving	Discuss coping strategies (Essex et al., 1999 [84];)^a^ Learn problem-solving method Problem-solve stress management	Learn about supportive family climates and coping ([6] [84]; [32];; Orsmond et al., 2006)^a^
Group meeting 4	Planning for independence	Learn about the importance of planning out and practicing tasks that are important for independent living	Learn about challenges to independence and strategies for supporting independence
Group meeting 5	Employment	Learn about job behaviors ([90, 92]; Taylor & Mailick, 2014)^a^ Problem-solve difficulties on the job	Learn about employment services and supports (Taylor & Seltzer, 2012 [92];; Taylor & Mailick, 2014)^a^ Discuss advocacy strategies
Group meeting 6	Community and relationships	Learn social planning and conversation strategies Problem-solve social difficulties	Finding community activities and social opportunities ([27] [68] [95];);^a^
Group meeting 7	Personal safety	Discuss communication skills and areas of difficulty. Talk about communication and why it is important to safety Problem-solve safety concerns	Receive information on long-term planning: powers of attorney, wills, trusts [29, 46]^a^ Discuss safety concerns for adults with ASD
Group meeting 8	Health and well-being	Learn about health and well-being during adulthood ([27]; Kring et al., 2010; Lainhart & Folstein, 1994)^a^	Learn about risks to parental health and well-being ([1]; Barker et al., 2010 [47];; Seltzer et al., 2010 [86];)^a^

^a^Sessions based on published findings of our research group

During each session, new material was often first conveyed in a didactic fashion through PowerPoints and/or handouts. After reviewing the materials, adults with autism were given the opportunity to practice using the materials in a way that was directly related to their individual goals and situation, either with each other or with a facilitator, and ask questions. For example, participants might identify a goal related to reaching out to a local company that was hiring, and then they could work to develop a script that they could use during a phone call with an interviewer. Similarly, participants might identify a goal of getting together with friends or other group members; during the practice time they would work to write down specific steps, create scripts for phone calls or texts, and agree to next step. Task analysis, modeling, and role-playing approaches were used as appropriate. At the end of each session, participants were assigned “homework” assignments, such as working toward next steps to reach personal goals or practicing skills learned in session. Participants were able to share about their progress at the start of the next session if they were comfortable. The group was encouraged to also share and/or practice their problem-solving skills based on what happened during the week.

Following completion of the 8 weekly group sessions, a series of 3 monthly group boosters (1.5 h each) were conducted for adults with ASD and parents (again meeting separately in the same building). Sessions provided an opportunity for group members to connect and to provide updates on progress with goals. For both adults and parents, boosters began with a period of “check ins” for updates on the adult’s and family’s functioning, followed by group problem-solving around emergent concerns. For both parents and adults, positivity and problem-solving were explicitly modeled and emphasized across all sessions.

We note that several steps were taken to ensure treatment fidelity. All intervention staff participated in university research ethics training, CITI human subjects training, and 16 total hours of in-person training to learn about study goals and procedures, review curriculum components (which were manualized and included fidelity checklists), and role-play conducting joining sessions and multi-family group sessions. Fidelity checklists (Additional File 1) were completed by intervention staff at each intervention session to support adherence to specific fidelity criteria. Following formal training, intervention staff met with the project PIs to review materials, practice aspects of the intervention, and receive feedback. Parent groups were facilitated by one PhD-level psychologist and one graduate student; the adult groups were facilitated by a Masters-level psychologist or social worker and one or more postdoctoral fellow and/or graduate students. At the conclusion of each intervention session, the supervising psychologist led the other intervention staff in a short debriefing meeting so that staff could express reactions, ask questions, and problem-solve any issues that may have arisen during either the parent group or the young adult group. Group facilitators also had supervision meetings twice a month to discuss field notes, troubleshoot problems, review fidelity checklists, review resources and referrals for families, and receive constructive feedback. This type of continuous staff training and supervision has been supported as an important mechanism for treatment fidelity [10]. Tracking of these fidelity checklists revealed 91% implementation fidelity.

### Measures

#### Autism symptoms and phenotype

As mentioned above, the current study used the CARS-2 [81], the WASI-II [100], and the Waisman Activities of Daily Living [54] to describe the sample, as well as the SCQ-Lifetime [72] to confirm ASD. The CARS-2 was completed by clinical research staff and includes 15 items on key behaviors related to autism diagnosis, each rated on a 7-point scale with higher scores indicating greater severity. The WASI-II is an individually administered assessment of intelligence, suitable for individuals aged 6–90 years of age. It was administered to the adults with ASD and provided full-scale IQ score estimates. Using the Waisman Activities of Daily Living, parents rated their son or daughter’s level of independence on 17 items covering the domains of personal care, housekeeping, and meal-related activities. Each item was rated on a 3-point scale and items were summed with higher scores indicating higher independence. Finally, the SCQ-lifetime is a 40-item caregiver-report screener for ASD. It has been comprehensively evaluated and is broadly used in research and practice. For the purposes of the current study, these measures were not further used in any analyses related to the current study’s research questions.

#### Engagement in work

At each time point, the adults with ASD were interviewed and were asked about their engagement in work-related activities. Specifically, the adults were asked the open-ended question: “Thinking back over the past month, have you done any kind of job or career-related activity?” Two coders on the research team independently coded adults answers into six work categories: 0 = *no work activities*; 1 = *minimal job exploration* (one activity such as “completing applications” or “researching online”); 2 = *diverse job exploration* (more than one activity such as “meeting with job coach to work on interview skills” and “attending job fairs”); 3 = *working for pay 1 or 2 times/week*; 4 = *working for pay 3 or 4 times/week*; and 5 = *working for pay 5 or more times/week*. In addition, individuals who were working (i.e., work engagement coded as 3–5), were given an additional point on the scale if they received recognition or took on additional roles and responsibilities at work, resulting in a possible range of 0–6 on the work engagement scale. Examples of activities resulting in an extra point included being a trainer of other employees, getting a raise, learning new tasks (e.g., started as bagger but now also works as cashier), and taking on additional work with another employer (e.g., reached max hours with one employer so getting more hours with second job). Having the additional point was a way to capture the upward mobility of some of the young adults even if they were not increasing in total number of hours worked. As an example, in the course of the study, one young adult had been promoted to an assistant manager position even though he was not working full-time. The additional point thus allowed for a way to provide “credit” for the professional growth and maturity. Inter-rater reliability for this 7-point work engagement variable was high (*ĸ* = 0.88).

Parents also separately reported on whether or not their son or daughter had worked for pay in the past week. Responses of 0 reflected “no work for pay” and 1 reflected “any work for pay.” Previous work with similar populations has indicated that parent-report of work for pay is a reliable measure in samples of adults with ASD and their parents (e.g., [89]). Parent ratings of work-for-pay were highly correlated with the coded adult ratings of work engagement (*rs* = .79–.92). In the present study, parent-report was examined within follow-up sensitivity analyses (see Additional File 2).

#### Engagement in meaningful activities

Adults with ASD also reported on their engagement in meaningful activities at each wave of data collection using the following item measured on a 5-point Likert scale, “how often do you do things that make you happy or proud?”, with response options ranging from 0 = never to 4 = all of the time. After we asked the adult with ASD to indicate how often they did something that made them happy/proud (frequency), we allowed them to elaborate on what makes them happy/proud in an open-ended way. The intention of this measure was to capture an individual’s engagement in activities that reflect their own values (i.e., an outcome that mattered to them). Participants were prompted to specify what activities they were involved in that resulted in positive mood, if that language was helpful to them. Our previous work has indicated that this 1-item measure has strong face validity and is sensitive to change in samples of families of individuals with ASD [86]. Examples of meaningful activities reported by the adults with ASD included having an art piece in a gallery, completing a distance race, doing chores and taking care of their cat, and finishing a woodworking project.

#### Behavior problems

Behavior problems were measured at each time point using the Problem Behavior subscale of the Scales of Independent Behavior-Revised (SIB-R [12];). Parents indicated the presence of behavior problems across three domains: internalized (hurtful to self, unusual or repetitive habits, withdrawn or inattentive behavior), externalized (hurtful to others, destructive to property, disruptive behavior), and asocial (socially offensive and uncooperative behavior). Each type of behavior problem was coded as manifested (1) or not manifested (0) during the past month. The *total number of types of behavior problems* (0–8) was calculated by summing across all eight categories of behavior problems. Next, parents who indicated that their son or daughter displayed a given category of behavior problem during the past month then rated the frequency of the behavior, from 1 = *less than once a month* to 5 = *one or more times*/*hour*, and the severity of the behavior, from 1 = *not serious* to 5 = *extremely serious*. Standardized algorithms [12] were used to translate the frequency and severity ratings into the following subscales: internalizing problems, externalizing problems, and asocial problems. Reliability and validity have been established by Bruininks et al. [12]. Higher values indicate higher levels of behavior problems and scores of 110 or higher reflect a clinically significant level of behavior problems.

#### Data analysis

In separate analyses, dependent variables included engagement in work, engagement in meaningful activities that made the adult feel happy and proud, and behavior problems (the number of types of behavior problems, internalizing behaviors, externalizing behaviors, and asocial behaviors). For our first research question, we conducted a series of three (time) by two (group) repeated measures ANOVAs to test for differences in change in the above outcomes of interest from pre-intervention to post-intervention (time 1, time 2, and time 3, respectively) between the intervention group and the control group. For our second question related to maintenance of treatment effects, we conducted a series of repeated measures ANOVAs to test for the within-group change in the outcome variables in the intervention group during the 6 months following the completion of the intervention (time 3, time 4, and time 5 waves of data collection). To answer our third research question related to replication of the treatment effect in the control group, we conducted a series of repeated measures ANOVAs to test for change in the outcome variables of interest in the control group during the period when they were receiving the intervention (time 3, time 4, and time 5 waves of data collection). We note statistical significance when *p* values are less than .05 for our first two outcomes of interest: engagement in work and engagement in meaningful activities. Additionally, since we utilized 4 different measures of behavior problems (i.e., number of types of behavior problems, internalizing problems, externalizing problems, and asocial problems), based on recommendations of Benjamini et al. [9], we utilized a sequential approach to controlling the false discovery rate and establishing the alpha value. Using total number of types of behavior problems (a global measure) as our primary endpoint within this family of tests, we set statistical significance for all behavior problem tests at *p* = .025 (i.e., number of types of behavior problems = .027 ≤ 2/4 (0.05) = .025). We also report means, standard deviations, and clinical cut-points for descriptive purposes.

## Results

### Research question 1: primary test of treatment effect

**Fig. 2:**
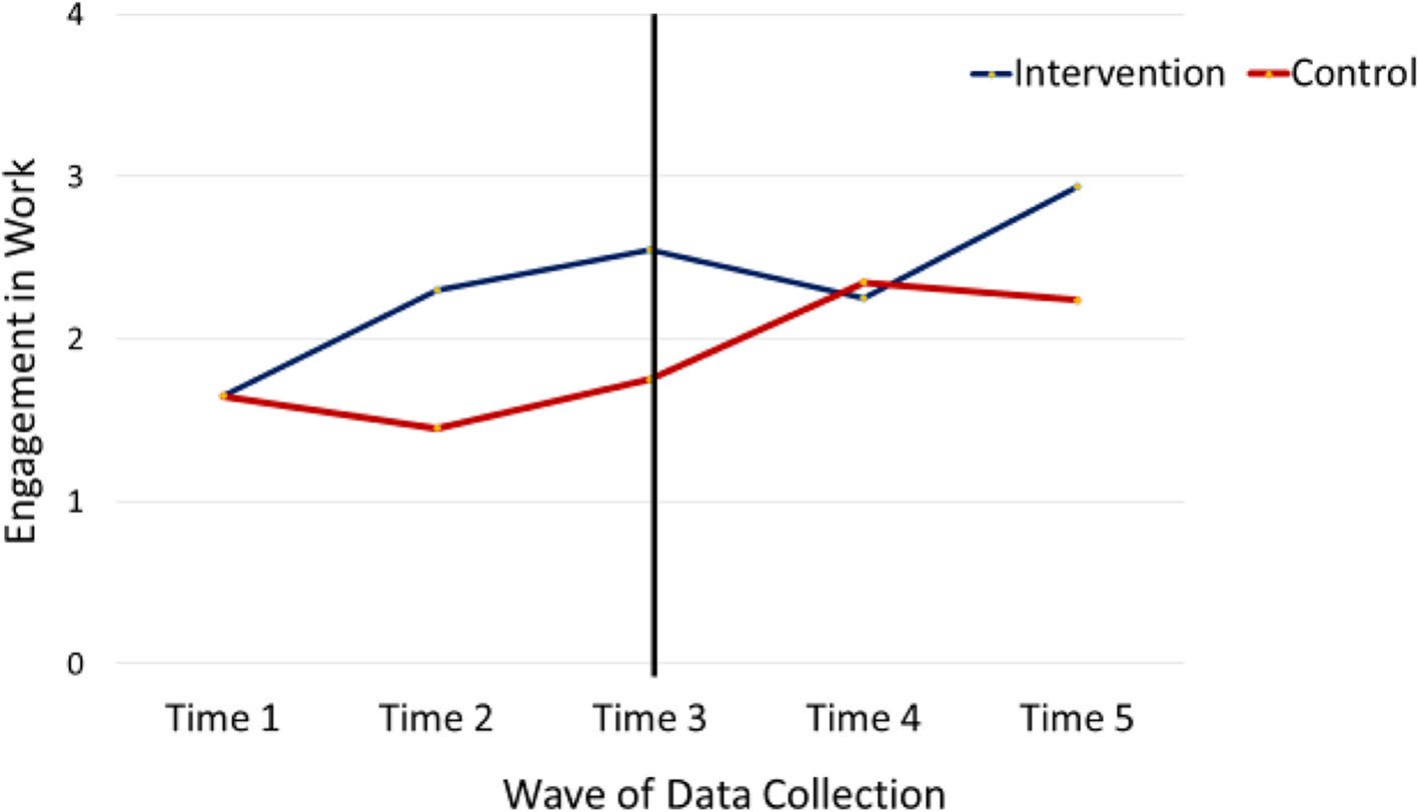
Engagement in work over time

To answer our first research question, we examined differences in change between the two groups from pre-intervention to post-intervention (times 1, 2, and 3) on engagement in work, engagement in meaningful activities, and behavior problems. Shown in Table 3 are means and standard deviations for both groups over time. Results for the interaction of group by time for engagement, including effect sizes, are also shown in Table 3. Based on self-reported engagement in work activities, there was an increase in work-related activities for the intervention group over the 6-month intervention period (*M* = 1.65, 2.30, and 2.55 at time 1, 2, and 3 respectively), compared to no change in work in the control group (*M* = 1.65, 1.45, and 1.75 at time 1, 2, and 3 respectively), *F* = 3.22, *p* = .081 (see Fig. 2, noting times 1–3 for both groups), although this was not statistically significant at the *p* = .05 level. Descriptively, the change in work-related activities for the intervention group over the 6-month period means that participants moved, on average, from “minimal to diverse job exploration” before intervention (1.65 is between a 1 = minimal job exploration and a 2 = diverse job exploration) to “diverse job exploration and working for pay 1-2 times per week,” on average, after intervention (2.55 is between a 2 = diverse job exploration and a 3 = working for pay 1–2 times per week). Further, a difference of one-half of a standard deviation has been used to indicate clinically significant change [77]. The standard deviation in engagement in work in the overall sample at baseline was 1.78 (*M* = 1.65, range = 0 to 5), indicating that the almost 1-point change in work engagement over the 6-month period, though not statistically significant at the *p* < .05 level, represents a clinically meaningful difference in work-related activities.

**Table 3:** Change from baseline to 6 months for intervention and control

	Intervention, ***n*** = 20			Control, ***n*** = 20			Group by time linear ***F*** and partial eta squared
	Time 1	Time 2	Time 3	Time 1	Time 2	Time 3	
Engagement in work activities	1.65 (1.66)	2.30 (1.78)	2.55 (1.76)	1.65 (1.93)	1.45 (1.93)	1.75 (1.92)	*F* = 3.22, *p* = .081, eta = .078
Engagement in meaningful activities	2.40 (.19)	2.60 (.20)	3.0 (.20)	2.45 (.18)	2.40 (.20)	2.30 (.20)	*F* = 8.43, *p* = .006, eta = .182
Number of type of behavior problems	3.65 (1.81)	2.75 (1.77)	2.65 (1.79)	3.55 (1.73)	3.30 (1.95)	3.45 (1.79)	*F* = 5.33, *p* = .027, eta = .123
Internalizing problems	117.00 (9.95)	108.70 (8.04)	109.60 (8.39)	111.95 (7.72)	111.45 (10.41)	110.85 (7.52)	*F* = 7.69, *p* = .009, eta = .168
Externalizing problems	102.10 (6.41)	100.45 (5.90)	100.65 (5.94)	102.70 (7.64)	100.70 (5.03)	100.60 (6.26)	*F* = 0.13, *p* = .716, eta = .004
Asocial problems	110.65 (11.05)	105.80 (10.53)	106.30 (11.84)	111.80 (11.45)	110.70 (10.14)	110.65 (9.14)	*F* = 1.42, *p* = .241, eta = .036

Importantly, a follow-up analysis using parent-reported work for pay (yes/no) similarly revealed a pattern of increase in work for pay for the intervention group over the 6-month intervention period, compared to the control group, although this was not statistically significant at the *p* = .05 level. These results are presented in Additional File 2 in Additional Table 1.

Also shown in Table 3, there was a statistically significant group by time interaction for engagement in meaningful activities, *F* = 8.43, *p* = .006. Adults in the intervention group reported a higher frequency of engaging in activities that made them feel happy/proud at post-intervention than at pre-intervention; a significant change was not observed in the control group. Descriptively, by the end of the 6-month period, 70% of the intervention group reported engaging in meaningful activities most or all of the time, compared to only 45% of those in the control group.

**Fig. 3:**
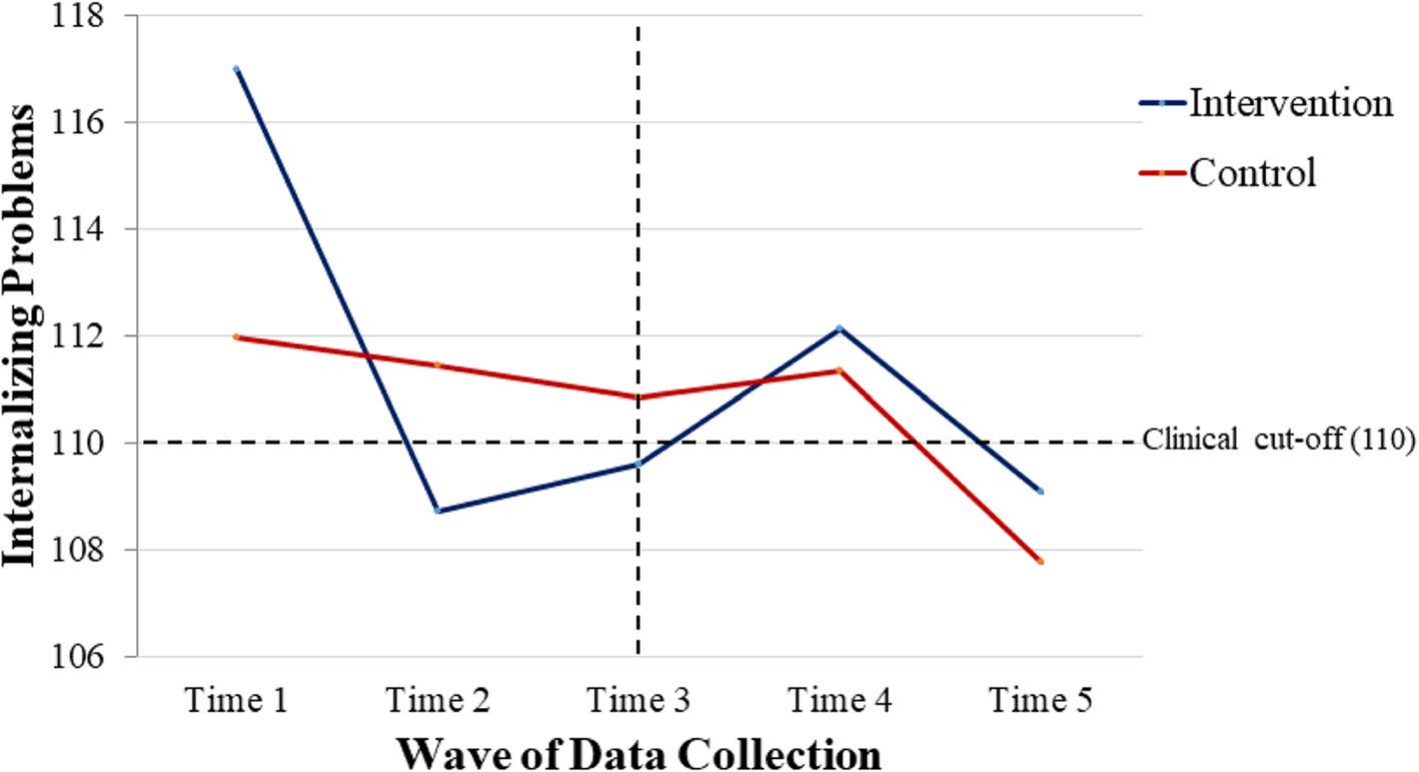
Internalizing problems over time

As shown in Table 3, there was a group by time interaction for total number of types of behavior problems, *F* = 5.33, *p* = .027 such that adults in the intervention group had a decreased number of behavior problems over time, whereas there were no differences over time for the control group. Notably, for adults in the intervention group, there was an average reduction of one type of behavior problem over the 6-month period from before to after the intervention. When examining the subscale scores for specific behavioral domains, there was a statistically significant group by time interaction for internalizing problems, *F* = 7.69, *p* = .009, such that adults in the intervention group had a decreased level of internalizing behaviors from pre- to post-intervention, whereas there was no change for the control group (see Fig. 3, noting times 1–3 for both groups). For descriptive purposes, we note that the clinical cut point for problem behavior on the SIB-R is 110; the mean score for the intervention group at baseline (*M* = 117.00) was well above that cut point. However, the mean for the intervention group at post-intervention (*M* = 109.60) had dropped below the clinical cut-off. For externalizing problems, mean scores at baseline were not clinically elevated for either group, and for asocial problems, mean scores at baseline were just at clinical cut offs for both intervention and control groups. The interaction terms for externalizing problems and asocial problems were not statistically significant, suggesting no statistically significant differences between the groups in change from pre- to post-intervention.

### Research question 2: maintenance of treatment effect in intervention group

To answer our second research question, we tested for maintenance of treatment effects by examining engagement in work, engagement in meaningful activities, and behavior problems during the 6-month period following completion of the intervention (time 3, time 4, and time 5 data collections). This analysis was restricted to intervention group cases with data at these waves of data collection (*n* = 16 out of 20 families). Means, standard deviations, and ANOVA results for all measures are shown in Table 4. Consistent with our hypothesis, we found maintenance of treatment effects in the 6 months after the end of the intervention, as evidenced by no statistically significant effects of time for any of the outcome measures. A follow-up analysis using parent-reported work for pay status (yes/no) provided similar results (see Additional File 2, Additional Table 2).

**Table 4:** Intervention cases from 6 to 12 months (*N* = 16)

	Time 3	Time 4	Time 5	ANOVA
				F and partial eta squared
Engagement in work activities	2.31 (1.70)	2.25 (1.88)	2.94 (2.08)	*F* = 2.25, *p* = .155, eta = .130
Engagement in meaningful activities	3.06 (.93)	2.88 (1.03)	3.06 (1.0)	*F* = 0.00, *p* = 1.00, eta = .00
Number of type of behavior problems	2.63 (1.89)	2.89 (1.78)	3.06 (1.57)	*F* = 1.35, *p* = .263, eta = .083
Internalizing problems	108.38 (8.30)	112.13 (8.84)	109.06 (7.68)	*F* = 0.16, *p* = .699, eta = .010
Externalizing problems	101.19 (6.43)	100.69 (6.53)	100.94 (7.51)	*F* = 0.05, *p* = .821, eta = .004
Asocial problems	105.81 (12.01)	105.13 (9.81)	106.25 (9.44)	*F* = 0.02, *p* = .891, eta = .001

### Research question 3: replication of treatment effect in control group

To answer our third research question regarding replication of the treatment effect in the control group, we tested for change in engagement in work activities, engagement in meaningful activities, and behavior problems from pre-intervention, mid-point, and post-intervention (time 3, time 4, and time 5 data collections). Individuals who were initially assigned to the control group received the intervention immediately after time 3; analysis was restricted to those cases with data at times 3, 4, and 5 (*n* = 17 out of 20 families). Means, standard deviations, and ANOVA results for all measures are shown in Table 5. There was a pattern of increase in engagement in work activities; however, the effect of time was not statistically significant. A follow-up analysis using parent-reported work for pay status (yes/no) provided similar results (see Additional File 2: Additional Table 3). Next, there was a statistically significant increase in engagement in meaningful activities, *F* = 5.76, *p* = .029. Regarding behavior problems, we observed a reduction in the number of types of behavior problems, although this was not significant at the adjusted *p* value of .025. However, the decrease in the level of internalizing problems (*F* = 6.53, *p* = .021) over time was statistically significant at the adjusted *p* < .025 level. Importantly, the internalizing mean score immediately before receiving the intervention was above the clinical cut point (*M* = 111.88) the mean was below the clinical cut point after the intervention (*M* = 107.76). There were no statistically significant effects of time for externalizing behavior problems, which were below the clinical cut point before and after the intervention, or for asocial behavior problems which were at or just below the clinical cutoff before and after the intervention. Consistent with our hypothesis, these intervention findings with the waitlist control group cases mirror the effect found for the intervention group when they received the intervention, reflecting replication of the treatment effect in a second cohort.

**Table 5:** Control cases from time 3 to time 5 (*n* = 17)

	Time 3	Time 4	Time 5	ANOVA F and partial eta squared
Engagement in work activities	1.71 (1.99)	2.35 (2.23)	2.24 (2.05)	*F* = 1.12, *p* = .306, eta = .065
Engagement in meaningful activities	2.24 (.90)	2.71 (.69)	2.59 (.80)	*F* = 5.76, *p* = .029, eta = .265
Number of type of behavior problems	3.65 (1.87)	3.41 (1.37)	2.94 (2.14)	*F* = 3.43, *p* = .083, eta = .176
Internalizing problems	111.88 (7.17)	111.53 (9.07)	107.76 (6.79)	*F* = 6.53, *p* = .021, eta = .290
Externalizing problems	100.88 (6.67)	101.47 (8.17)	100.12 (5.15)	*F* = 0.69, *p* = .417, eta = .041
Asocial problems	110.53 (9.83)	109.42 (8.54)	108.94 (13.24)	*F* = 0.56, *p* = .466, eta = .033

## Discussion

Using a randomized waitlist control design, the present study evaluated a multi-family group psychoeducation intervention, *Working Together,* for adults on the autism spectrum without ID. Consistent with results of multi-family group psychoeducation interventions for adolescents with ASD without ID [22, 83] as well as for individuals with mental health conditions (e.g., mood disorders [73];), our findings point to promising, important benefits of this intervention approach for adults with ASD. Specifically, in the present study, the *Working Together* intervention was associated with medium to large effect sizes associated with the *Working Together* intervention across key outcomes, including adults on the spectrum experiencing significant increases in meaningful activities and decreases in internalizing problems. Although increases in work-related activities were not statistically significant, an observed one half of a standard deviation difference from before to after the intervention indicated clinically significant change.

### Engagement in work

In the present study, we found a medium to large effect size for change in engagement in work activities over a 6-month period, with an almost 1-point change in work engagement on a 7-point scale observed for the intervention group. Though not statistically significant at the *p* < .05 level, based on a one-half of a standard deviation as a marker of clinically significant change (e.g., [77]), the change found for the intervention group represents a clinically meaningful difference in work-related activities. Participants moved, on average, from “minimal to diverse job exploration” before the intervention to “diverse job exploration and working for pay 1-2 times per week,” on average, after the intervention. However, overall employment was still relatively low (i.e., at 6-month follow-up, only 50% of adults in the initial intervention group had worked for pay in the past week [38% prior to intervention]). The extreme difficulty many adults with autism face in finding employment, maintaining employment, and experiencing upward career mobility is well-documented, with personal characteristics as well as environmental resources being associated with employment outcomes (e.g., Chan et al., 2017 [37, 41, 71, 78, 89, 91, 92];). The flexibility and fluidity often required to secure and maintain employment (e.g., adjusting approach to search based on the type of job; responding to differential expectations of multiple supervisors) are likely challenging for adults with autism at any IQ level. It is in this context that the current study’s finding should be interpreted, suggesting that movement into more diverse work exploration and limited paid employment is a particularly meaningful and important change for adults with ASD with high levels of disengagement.

### Engagement in meaningful activity

Although paid employment is one important index of engagement, less research has focused on the engagement of adults with ASD in activities that are of value to them and that they find to be meaningful to their lives, whether or not they are for pay [37]. Findings from the present study suggest that multi-family group psychoeducation may be one mechanism for increasing engagement in such activities, as we observed a large effect on meaningful activities that are important to adults with ASD. The loss of structure following high school exit has been posited as a risk for well-being for individuals with ASD [95]. Conversely, higher levels of community-based independence have been associated with improvements in behavioral functioning over time [92]. The *Working Together* intervention leveraged the goals and values of adults with ASD without ID and their families to deliver research-based education on key topics relevant to adulthood (e.g., employment, planning for independence, coping, community, and relationships; see Table 2) as well as training and practice in problem-solving around family-identified concerns to both adults with ASD and their parents. These intervention components enhanced specific skills, in combination with family support, for the adults to engage in consistent meaningful activities that brought personal happiness, broadening our understanding and definitions of “success” beyond solely paid activities. Interestingly, at the 6-month follow-up, individuals in the intervention group reported engaging in meaningful activities “most of the time,” on average, suggesting that these benefits were sustained.

### Behavioral functioning

In addition to the observed benefits of meaningful activities, participating in the *Working Together* intervention was associated with clinically meaningful reductions in behavior problems. Notably, we found a large effect of the intervention for the internalizing problem subscale of the SIB-R which is a measure of withdrawn/inattentive behavior and unusual/repetitive habits. These findings were very robust, as the improvements in internalizing problems remained over the 6-month follow-up period. Further, decreases in internalizing problems were observed for individuals in the waitlist group upon receiving the intervention, thus replicating the findings. Taken together, findings suggest a promising shift for individuals over the course of the intervention away from clinically interpreted internalizing behavior to more meaningful engagement in daily life.

### Limitations and future research directions

The present study was not without limitations. First, the sample size was small and adults in the study were primarily White. Parents also had relatively high levels of educational attainment, limiting the generalizability of the findings. Further, the intervention was focused on individuals with ASD who did not have ID and who at the time of enrollment were disengaged from vocational/educational activities (defined as less than 10 h/week engaged in employment or educational activities). More research is needed to extend this work and evaluate the multi-family group psychoeducation approach with families from culturally and linguistically diverse backgrounds, for those with more significant support needs, and for those with higher initial levels of engagement. We also recognize that although this study focused specifically on the direct effects of the intervention on adult engagement and behavior, it is likely that family stress and well-being, as well as the quality of family relationships, play a crucial role in intervention efficacy given the family-focused nature of the intervention approach. Future research should investigate how parent and relationships factors may influence or moderate the efficacy of the *Working Together* intervention.

There were also limitations related to measurement. Our measure of engagement with meaningful activities was based on a single Likert-scale item; future work should consider more thoroughly examining specific meaningful activities in which the adults were engaged. We also found that our coding scheme was sensitive to change; however, not all participants described the number of hours per shift and/or the number of jobs per day and week. As such, our measure was not well-suited to answer research questions about “dosage” of work-related activities or determine how much and what type of work activity is sufficient to foster self-efficacy and a sense of accomplishment; daily diary methodology may be useful for answering these types of questions in future research. Further, we did not gather data on satisfaction with work or the fit of the work with the goals and interests of the adult on the spectrum. We also did not ask the adults with autism, who were disengaged at the start of the study, to describe the specific challenges they face in the job search process or reasons they feel they were not more successful in securing work and career advancement. Additional research to understanding where break downs are happening in employment pathways (i.e., not getting interviews and/or job offers; only being hired for a few hours or hired for temporary jobs) would be valuable. Partnering with adults with autism in this future work will be critical to best inform research question development and data collection, analysis, and interpretation.

Findings from the present study also suggest a need for further research to understand how the intervention may need to be adapted or coupled with other services to maximize benefits. The present study only followed families in the intervention for 6 months after completion of the intervention. Future work should examine if effects of the intervention are sustained for a longer duration and what forms of supports may be needed to maintain gains over time. Also, for adults with autism who desire full-time employment, it may be that they need a different dosage of *Working Together* (increased intensity and/or duration), additional forms of treatment/intervention, and/or policies that promote diverse integrated employment opportunities in order to best support sustained employment. Given the meaningful change experienced by participants who received *Working Together*, continuing to regularly receive the intervention could help further engage adults with ASD in paid and non-paid activities and support them in navigating any emergent difficulties through the problem-solving process. It also may be useful to consider pairing*Working Together* with other evidence-based practices. For example, individuals with ASD could benefit from very targeted intervention to improve their job interviewing skills [87]. Adults with ASD may have more success in securing and maintaining employment if the *Working Together* intervention was paired with intensive employment support and if staff at employment sites receive support and training on how to best support the adult with ASD to be successful in their workplace. It will be important for future research to answer questions regarding how to tailor dosage, timing, and combination of treatments and services to enhance intervention effects.

Juxtaposed with these limitations are several strengths. The study included reports provided by both adults with ASD and their parents. Consistent with calls for a more holistic conceptualization of outcomes during adulthood [94], adults with ASD reported on their engagement in meaningful activity, highlighting the intervention’s positive effects on an outcome that mattered specifically to the person on the autism spectrum. Further, we employed an iterative process of intervention development, drawing from our longitudinal research findings and gathering input directly from adults on the spectrum and their families through focus groups, our advisory board, and research team members with lived experience; this helped to ensure the relevance of the curriculum for the end users. Next, the study design allowed for testing of maintenance effects 6 months after the end of the intervention as well as replication of the treatment effects in a separate cohort (i.e., the waitlist control group). Taken together, these findings suggest medium to large effects of the intervention. Finally, women are often underrepresented in studies of adults with ASD and it is also notable that over a third of the individuals with ASD in the current study were female or nonbinary.

## Conclusions

In conclusion, results from this randomized waitlist control trial highlight potential benefits of the *Working Together* program for improving engagement in both work-related and other meaningful activities, as well as reducing internalizing problems, for adults with ASD. These findings are consistent with the gains demonstrated in studies of multi-family group psychoeducation interventions conducted with other populations [24, 59]. Addressing calls for more intervention services research during adulthood (e.g., US Department of Health and Human Services’ Interagency Autism Coordinating Committee strategic plan), and specifically the need for family-centered supports [66], *Working Together* is a promising multi-family group psychoeducation intervention designed to support adults on the autism spectrum and their families in order to improve functioning during adulthood.

## Supplementary Information

**Additional file 1.** Fidelity Checklist. Checklist completed by intervention staff at each intervention session to support adherence to specific fidelity criteria.

**Additional file 2.** Intervention effects on parent-reported work for pay status. Follow-up analyses of intervention effects using parent-reported work for pay status.

## Data Availability

The datasets generated during and/or analyzed during the current study are available from the corresponding author on reasonable request.
