# Supplementary material for: Impact of Working Together for adults with autism spectrum disorder: a multifamily group intervention
Source: J Neurodev Disord. 2021 Oct 8;13:44. doi: 10.1186/s11689-021-09395-w (PMC8499454; doi:10.1186/s11689-021-09395-w)
Supplement: Supplementary file 1 — Additional file 1. Fidelity Checklist. Checklist completed by intervention staff at each intervention session to support adherence to specific fidelity criteria. [file 11689_2021_9395_MOESM1_ESM.docx]

**Fidelity Checklist**

**Date : ___________ Session# : _________ Form Completed By: _____________________**

| **Activity** | **Score** |
| --- | --- |
| Welcome/Announcements: Field questions, affirm value of problem-solving process, review prior week | **2 1 0 NA** |
| Check-in | **2 1 0 NA** |
| Present content (e.g., slides, handouts) | **2 1 0 NA** |
| Review/reinforce problem-solving rules and principles | **2 1 0 NA** |
| Practice problem-solving | **2 1 0 NA** |
| Closing wrap-up, consider/discuss possible additional resources that may be needed based on group needs | **2 1 0 NA** |
| All surveys completed | **2 1 0 NA** |
| Use of facilitation behaviors (e.g., reframing, developmentally appropriate language, rapport building, praise, etc.) | **2 1 0 NA** |
| All engagement ratings completed | **2 1 0 NA** |
| Debrief with staff and plan for next week | **2 1 0 NA** |
| Mid-week check-ins from prior week | **2 1 0 NA** |
| Follow-up on specific concerns and questions for each member/family (If questions/concerns raised) | **2 1 0 NA** |

***** Scoring Key: 2 = Implemented; 1 = Partially Implemented; 0 = Did Not Implement; NA = Not Applicable***
