# Supplementary material for: Impact of Working Together for adults with autism spectrum disorder: a multifamily group intervention
Source: J Neurodev Disord. 2021 Oct 8;13:44. doi: 10.1186/s11689-021-09395-w (PMC8499454; doi:10.1186/s11689-021-09395-w)
Supplement: Supplementary file 2 — Additional file 2. Intervention effects on parent-reported work for pay status. Follow-up analyses of intervention effects using parent-reported work for pay status. [file 11689_2021_9395_MOESM2_ESM.docx]

Additional Table 1. Change in parent-reported work-for-pay status from Baseline to 6 months for Intervention and Control

|  | **Intervention n=20** | | | **Control n=20** | | | **Group by Time Linear F and partial ⴄ^2^** |
| --- | --- | --- | --- | --- | --- | --- | --- |
|  | Time 1 | Time 2 | Time 3 | Time 1 | Time 2 | Time 3 |  |
| Engagement in work  (% working for pay) | .30 (.47)  (30%) | .45 (.51)  (45%) | .45 (.51)  (45%) | .40 (.50)  (40%) | .35 (.49)  (35%) | .30 (.47)  (30%) | F=3.74, *p*=.061, eta=.090 |

Additional Table 2. Change in parent-reported work-for-pay status in Intervention Cases from 6 to 12 months (N=16)

|  |  | | | **ANOVA** |
| --- | --- | --- | --- | --- |
|  | Time 3 | Time 4 | Time 5 | **F and partial ⴄ2** |
| Engagement in work  (% working for pay) | .38 (.50)  (38%) | .31 (.48)  (31%) | .50 (.52)  (50%) | F=2.14, *p*=.16,  eta=.125 |

Additional Table 3. Change in parent-reported work-for-pay status in Control Cases from Time 3 to Time 5 (n=17)

|  |  | | | **ANOVA** |
| --- | --- | --- | --- | --- |
|  | Time 3 | Time 4 | Time 5 |  |
| Engagement in work  (% working for pay) | .29 (.47)  (29%) | .35 (.49)  (35%) | .35 (.49)  (35%) | F=.32, *p*=.58,  eta=.02 |
